# Novel dimeric DOTA-coupled peptidic Y_1_-receptor antagonists for targeting of neuropeptide Y receptor-expressing cancers

**DOI:** 10.1186/2191-219X-1-21

**Published:** 2011-09-02

**Authors:** David Chatenet, Renzo Cescato, Beatrice Waser, Judit Erchegyi, Jean E Rivier, Jean Claude Reubi

**Affiliations:** 1 The Clayton Foundation Laboratories for Peptide Biology, The Salk Institute for Biological Studies, 10010 N. Torrey Pines Rd., La Jolla, CA, USA; 2 Institut National de la Recherche Scientifique, Laval, QC, Canada; 3 Division of Cell Biology and Experimental Cancer Research, Institute of Pathology, University of Berne, PO Box 62, Murtenstrasse 31, CH-3010 Berne, Switzerland

**Keywords:** neuropeptide Y receptor, tumor imaging, oncology, peptide receptor radionuclide therapy, breast cancer, antagonist

## Abstract

**Background:**

Several peptide hormone receptors were identified that are specifically over-expressed on the cell surface of certain human tumors. For example, high incidence and density of the Y_1 _subtype of neuropeptide Y (NPY) receptors are found in breast tumors. Recently, we demonstrated that the use of potent radiolabeled somatostatin or bombesin receptor antagonists considerably improved the sensitivity of *in vivo *imaging when compared to agonists. We report here on the first DOTA-coupled peptidic Y_1 _receptor affine dimer antagonists.

**Methods:**

Based on a Y_1 _affine dimeric peptide scaffold previously reported to competitively antagonize NPY-mediated processes, we have developed new dimeric DOTA-coupled Y_1 _receptor affine antagonists for scintigraphy and radiotherapy. These dimeric peptides were tested for their specific binding to Y_1 _expressed in SK-N-MC cells and Y_2 _expressed in SH-SY5Y as well as for their ability to mediate cAMP production in SK-N-MC cells.

**Results:**

Introduction of two DOTA moieties at the N-termini of the dimeric NPY analogs as well as the double Asn^29 ^replacement by Dpr(DOTA) or Lys(DOTA) (**6 **and **10**) moiety dramatically reduced binding affinity. However, asymmetric introduction of the DOTA moiety in one segment of the peptidic heterodimer (**8 **and **11**) resulted in suitable antagonists for receptor targeting with high binding affinity for Y_1_. All compounds were devoid of Y_2 _binding affinity.

**Conclusions:**

The design and the *in vitro *characterization of the first DOTA-coupled dimeric NPY receptor antagonist with high affinity and selectivity for Y_1 _over Y_2 _are described. This compound may be an excellent candidate for the imaging of Y_1_-positive tumors and their treatment.

## Background

Peptide hormone receptors play an increasing role in cancer medicine. This role is based primarily on the peptide receptor over-expression on tumor cells which allows a specific receptor-targeted scintigraphic tumor imaging and tumor therapy with radiolabeled peptide analogs [1]. The somatostatin receptors were the first peptide receptors identified for these purposes, and somatostatin receptor targeting has now become an integral part of the routine management of patients with gastroenteropancreatic neuroendocrine tumors. Somatostatin receptor scintigraphy (OctreoScan^®^, Covidien Ltd., St. Louis, MO, USA) detects these tumors with extremely high sensitivity and specificity [2]. Moreover, recent results from clinical studies involving somatostatin receptor radionuclide therapy of these tumors are very promising [2]. The last decade has seen the development of numerous novel somatostatin agonists suitable for tumor targeting [3,4]. Interestingly, however, it has recently been shown that potent somatostatin receptor antagonists, known to poorly internalize into tumor cells, can visualize tumors *in vivo *as well, or even better than the corresponding agonists [5]. This unexpected phenomenon was found both for sst_2_- and sst_3_-selective somatostatin analogs, and may be due to the binding of the antagonist to a larger number of sites and to its lower dissociation rate. A pilot clinical trial with radiolabeled DOTA-linked sst_2 _antagonists recently confirmed the animal data [6].

Prompted by the success of somatostatin receptor targeting, the over-expression of other peptide receptor families was evaluated in tumors *in vivo *[1]. Promising new candidates for *in vivo *peptide receptor targeting of tumors are neuropeptide Y (NPY) receptors, based on their high expression in specific cancers, in particular breast carcinomas [7,8]. In humans, four NPY receptor subtypes exist, called Y_1_, Y_2_, Y_4_, and Y_5 _[9]. The natural ligands for these receptors are the peptides of the NPY family, including the neurotransmitter NPY and the two gut hormones peptide YY (PYY) and pancreatic polypeptide (PP). Via binding to the NPY receptors, these peptides regulate a wide variety of physiologic functions such as digestion, vasoconstriction, and reproduction. They also play a key role in eating behavior [10]. On this basis, Y_2 _and Y_4 _receptor agonists and Y_1 _and Y_5 _receptor antagonists have become potential drugs against overweight that are currently evaluated for this application [11]. On the other hand, since Y_1 _and Y_2 _receptors are highly over-expressed in breast cancer, Ewing sarcomas, neuroblastomas, and high-grade gliomas [7,12-14], the use of radiolabeled Y_1 _and Y_2 _receptor ligands for an NPY receptor-targeted imaging and radiotherapy of these tumors, was suggested [1,15]. It is worth mentioning that in those tumors, the presence of a significant amount of Y_4 _or Y_5 _receptors was not observed [8]. A daunorubicin-coupled cytotoxic NPY analog [16], a Y_2_-selective, ^99 m^Tc-labeled radioactive NPY analog [17], and more recently, a ^99 m^Tc-labeled Y_1 _agonist were developed [18]. Preliminary clinical data on Y_1_-targeted tumor imaging with the latter compound in breast cancer patients are encouraging [18].

There is no information yet on Y_1 _receptor antagonists coupled to a chelator, which are suitable for *in vivo *receptor targeting. The aim of the present study was to design and develop dimeric NPY analogs coupled to the DOTA chelator for suitable radiolabeling that could be used for imaging and for radiotherapy of Y_1_-expressing tumors. We report here on the first dimeric DOTA-coupled peptidic NPY receptor antagonists with high affinity for Y_1_.

## Methods

### Reagents

All Boc-N^α^-protected amino acids with side chain protection: Arg(Tos), Asn(Xan), Cys(Acm), Tyr(2-Br-Z) were commercially available (Bachem Inc., Torrance, CA, USA). All reagents were of best grade available and were purchased from common suppliers. Silver trifluoromethanesulfonate was obtained from Sigma-Aldrich (St. Louis, MO, USA). DOTA-NHS was from Macrocyclics (Dallas, TX, USA). The adenylate cyclase activation flashplate assay (SMP004) was from PerkinElmer (Waltham, MA, USA).

### Peptide synthesis

**Figure 1:**
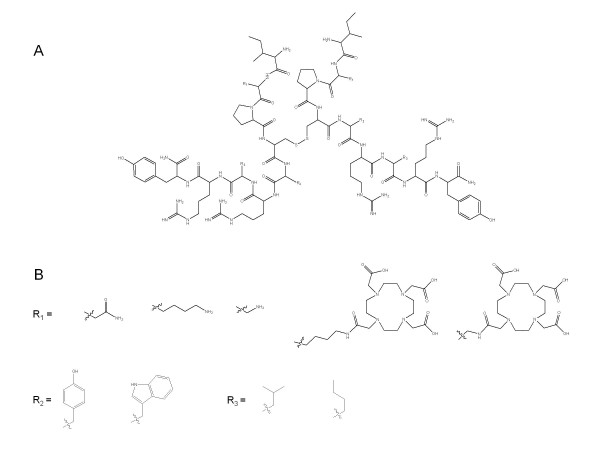
**Structure of the dimeric NPY scaffold and of the incorporated substitutions**. **(A) **Amino acid scaffold upon which NPY dimers are built; **(B) **structures of the substitutions incorporated at R_1 _to R_3_.

Each segment of the dimeric peptides was synthesized manually on a methylbenzhydrylamine (MBHA) resin using the solid phase approach and the Boc- strategy. Noteworthy, an orthogonally protected cysteine, i.e., Boc-Cys(Acm)-OH, was used to prevent dimerization of inadequate segment. Main chain assembly was mediated by diisopropylcarbodiimide (DIC) and coupling completion (45 to 60 min) was assessed by Kaiser's test. Threefold excess of protected amino acid and DIC was used based on the original substitution of the MBHA resin (0.4 mmol.g^-1^) and Boc removal was achieved *via *TFA-mediated deprotection (50% in dichloromethane (DCM); 10 to 15 min). An isopropyl alcohol (1% m-cresol) wash followed TFA treatment and then successive washes with triethylamine (TEA) solution (10% in DCM), methanol, TEA solution, methanol, and DCM completed the neutralization sequence. In order to facilitate the specific linkage of the DOTA moiety in the peptide dimers, the N-terminal amino acid of the sequence was introduced as an Fmoc derivative except when the DOTA moiety was coupled to the N-terminus. Peptide resins were then treated with anhydrous HF in the presence of anisole (5% to 10%, *v*/*v*) at 0°C for 1.5 h to liberate the Cys(Acm)-protected crude peptides. After elimination of HF under vacuum, the crude peptides were washed with peroxide-free diethyl ether and extracted with 0.1% TFA in 60% acetonitrile/water. After lyophilization, the orthogonally protected peptides were purified using preparative RP-HPLC and two successive solvent systems (eluent A: TEAP at pH 2.25 and 0.1% TFA, eluent B: 60% acetonitrile/A). The purified peptides were characterized by analytical RP-HPLC and MALDI-TOF-MS on a Voyager DE-STR in the reflector mode using the α-cyano-4-hydroxycinnamic acid as matrix. Conjugation of the DOTA derivative was achieved prior to the disulfide bond formation as previously reported [19]. Briefly, a solution of DOTA-NHS ester (2 eq) in DMF and N, N'-diisopropylethylamine (DIPEA) (3 eq) were added to the monomer solution in dry DMF. The mixture was stirred at room temperature and the progress of the reaction was followed by analytical RP-HPLC. After completion of the reaction, generally observed after 3 h, a preparative RP-HPLC purification was performed yielding the DOTA-conjugated analog. Homogeneity of each fraction was assessed by analytical RP-HPLC. Removal of the Acm group was achieved by silver trifluoromethanesulfonate (100 eq/Acm) treatment of each monomer (dissolved in TFA/anisole; 99:1, 1 mg/mL) at 4°C for 2 h. The peptide silver salt was then precipitated with diethyl ether and separated by centrifugation. Dimers were obtained by the treatment of two identical monomers (homodimer) or two different monomers (heterodimer) in equimolar concentration with aqueous 1 M HCl/DMSO (1:1) overnight at room temperature resulting in the removal of the silver ions as AgCl and disulfide bond formation. Following the filtration of silver chloride, dimeric peptides were once again purified and analyzed as described above. The structure of the dimeric NPY scaffold as well as the structure of the incorporated substitutions is shown in Figure 1.

### Cell lines

The neuroepithelioma cell line SK-N-MC endogenously expressing the NPY Y_1 _receptor was obtained from ATCC (HTB-10; LGC Standards, Teddington, Middlesex, UK) and cultured at 37°C and 5% CO_2 _in MEM with GlutaMax I and supplemented with 10% FBS, 100 U/ml penicillin and 100 μg/ml streptomycin, 1 mM MEM sodium pyruvate, and MEM non-essential amino acids (× 1). The neuroblastoma cell line SH-SY5Y endogenously expressing the NPY Y_2 _receptor, kindly provided by Dr. Paolo Paganetti (Novartis, Basel, Switzerland), was cultured at 37°C and 5% CO_2 _in MEM/Ham's F12 with GlutaMax I and supplemented with 10% FBS, 100 U/ml penicillin and 100 μg/ml streptomycin, 1 mM MEM sodium pyruvate, and MEM non-essential amino acids (× 1). All culture reagents were from Gibco BRL, Life Technologies, (Grand Island, NY, USA).

### Receptor autoradiography

Binding affinities of the compounds were assessed using sections of cell membrane pellets of SK-N-MC for Y_1 _or SH-SY5Y for Y_2_, as described before [7,13,20]. Briefly, membrane pellets were prepared and stored at -80°C, and receptor autoradiography was performed on 20-μm-thick cryostat (Microm HM 500, Walldorf, Germany) sections of the membrane pellets, mounted on microscope slides and stored at -20°C as previously described for other cell lines and receptors [21]. The slides were preincubated in Krebs-Ringer solution (NaCl 119 mM, KCl 3.2 mM, KH_2_PO_4 _1.19 mM, MgSO_4 _1.19 mM, NaHCO_3 _25 mM, CaCl_2 _2.53 mM, D-glucose 10 mM; pH 7.4) for 60 min at room temperature. Then, they were incubated for 120 min in the incubation solution containing the Krebs-Ringer solution, 0.1% BSA, 0.05% bacitracin, and 10,000 cpm/100 μl of the ^125^I-labeled human PYY (hPYY; 2,000 Ci/mmol; Anawa, Wangen, Switzerland). Membrane pellet sections were incubated with ^125^I-hPYY in increasing concentrations ranging from 0.1 nM up to 1,000 nM of non-labeled hPYY, as control, or with the compounds to be tested. After the incubation, the slides were washed two times for 5 min and then rinsed four times in ice-cold preincubation solution. After drying, the slides were exposed to Kodak films Biomax MR^® ^for 7 days. IC_50 _values were calculated after quantification of the data using a computer-assisted image processing system (Analysis Imaging System, Interfocus, Mering, Germany). Tissue standards (autoradiographic [^125^I] and/or [^14^C] microscales, GE Healthcare, Little Chalfont, UK) containing a known amount of isotope, cross-calibrated to tissue-equivalent ligand concentrations were used for quantification [22].

### Adenylate cyclase activity

Adenylate cyclase activity was determined in SK-N-MC cells using the adenylate cyclase activation flashplate assay (SMP004) from PerkinElmer (Waltham, MA, USA). SK-N-MC cells were seeded in 96-well culture plates at 25,000 cells/well and cultured for 48 h at 37°C and 5% CO_2_. Culture medium was then removed from the wells and fresh medium (100 μL) containing 0.5 mM 3-isobutyl-1-methylxanthine (IBMX) was added to each well. Cells were incubated for 30 min at 37°C. Medium was then removed and replaced with fresh medium containing 0.5 mM IBMX, with or without 10 μM forskolin and various concentrations of the peptides to be analyzed. Cells were incubated for 30 min at 37°C. After removal of the medium, cells were lysed and cAMP accumulation was determined using the SMP004 kit from PerkinElmer according to the instructions of the manufacturers.

## Results

### Rational design of dimeric Y_1 _receptor affine peptide antagonists

**Figure 2:**
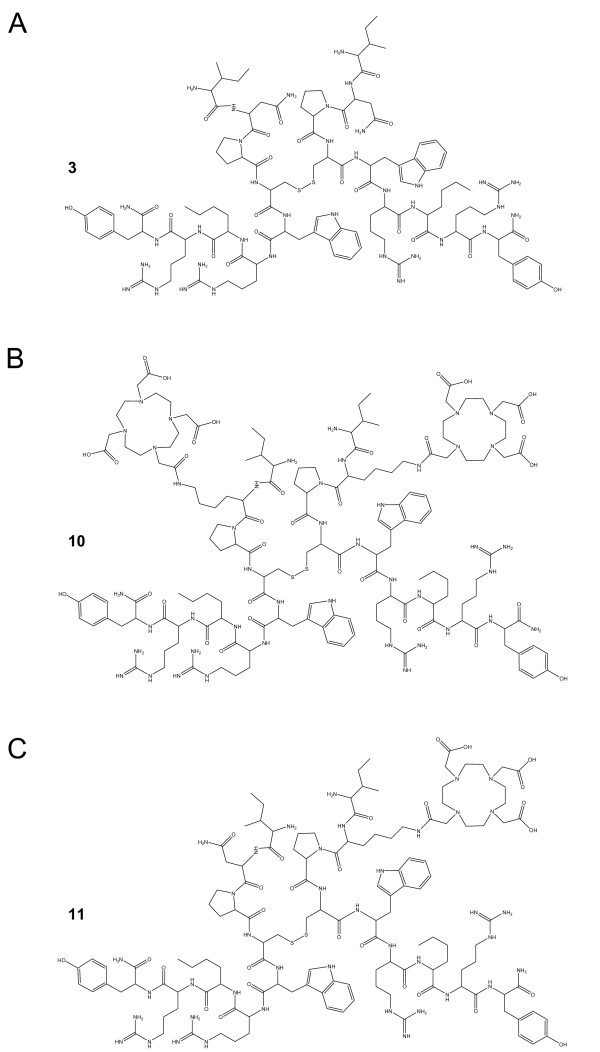
**Structure of the DOTA-free dimeric Y_1 _receptor affine antagonist (3) and its DOTA-conjugated dimeric counterparts**. Structure of (**A**) the DOTA-free dimeric Y_1 _receptor affine antagonist (**3**) and its DOTA-conjugated dimeric counterparts (**B**) **10 **and (**C**) **11**.

In order to generate and identify competitive and Y_1 _receptor affine antagonists suitable for breast cancer targeting, we took advantage of previously reported structure-activity relationship studies. Using a known dimeric Y_1_-selective antagonist (analog **1**, Table 1) as our template [23], we first looked at modifications supposedly increasing the antagonistic property and/or selectivity of NPY analogs. As a matter of fact, the replacement of Tyr^32 ^(NPY numbering) by a Trp and introduction of a hydrophobic and bulky residue such as norleucine (Nle) in position 34 were shown to increase Y_1 _receptor-binding affinity [24]. The DOTA-conjugated counterparts of peptides **1 **and **3 **(Table 1) were then obtained through the addition of the chelator moiety at the N-termini of the peptide dimers (analogs **2 **and **4**, Table 1). Other sites for the introduction of the chelating derivative were also considered. Based on previously reported structure-activity relationship studies, only the residue at position 29, i.e., Asn, was tolerant to amino acid replacement [25,26]. The C-terminal part of NPY, i.e., regions 32 to 36, is known to be directly involved in Y_1_-receptor binding [26,27] and was therefore not considered in our study. Similarly, the proline residue at position 30 is essential for the antagonistic behavior of such analogs and was consequently not replaced [28]. Nevertheless, we investigated the possibility to replace the proline residue by a cis or trans amino-proline moiety to which DOTA could be coupled. However, this substitution resulted in a significant reduction of binding affinity (data not shown). Thus, we replaced Asn^29 ^with a diaminopropionic (Dpr) (**5**) or a lysine (Lys) residue (**9**), and attached the DOTA moiety to the β or ε amino group of these residues, respectively. Analogs **6 **and **10**, with two chelators, exhibited lower binding affinities than their parent peptides (Table 2). These two amino acids, varying only by the number of carbon in their side chain, will also help us investigate the impact of the spatial proximity between the DOTA moiety and the peptide backbone on the biological activity. Addition of DOTA derivative was often followed by a concomitant reduction of binding affinity, most probably due to the steric hindrance of such residue, as exemplified with somatostatin analogs [29]. With no understanding on how the conjugation of the chelator on each segment of the peptide dimer will impact the binding affinity, selectivity, and biological activity, we investigated the possibility to generate heterodimer bearing only one DOTA derivative (analogs **8 **and **11**, Table 1) thus keeping the other peptide segment intact to ensure high binding affinity. Each purified monomer was usually obtained in a 15% to 25% yield calculated on the base of the substitution of the starting resin. After completion of the DOTA-conjugation and the removal of the Fmoc protecting group (if necessary), the purified monomeric DOTA-conjugated derivatives were generally obtained in a 25% to 30% yield based on the starting material. Finally, following the dimerization and the subsequent purification steps, the final products were isolated in a 10% to 15% yield based on the equimolar concentration of the starting materials. In total, 11 peptides were synthesized, purified and analyzed by RP-HPLC, CZE and MALDI-TOF spectrometry. Data are listed in Table 1. The structure of a DOTA-free homodimeric (**3**), a DOTA-conjugated homodimeric (**10**) and a DOTA-conjugated heterodimeric (**11**) Y_1 _receptor affine antagonist are shown in Figure 2.

**Table 1:** Physicochemical properties of free and DOTA-coupled dimeric peptide NPY analogs


Analog number	Amino acid residues				N-term	Purity (%)		MS^c ^	
	Xa^29^	Xa^29'^	Xb	Xc	DOTA	HPLC^a^	CZE^b^	calc	obs
**1**	Asn	Asn	Tyr	Leu	No	98	98	2,389.2	2,390.3
**2**	Asn	Asn	Tyr	Leu	Yes	97	96	3,161.6	3,162.6
**3**	Asn	Asn	Trp	Nle	No	94	90	2,435.2	2,436.5
**4**	Asn	Asn	Trp	Nle	Yes	95	92	3,207.6	3,208.8
**5**	Dpr	Dpr	Trp	Nle	No	99	96	2,379.3	2,380.1
**6**	Dpr(DOTA)	Dpr(DOTA)	Trp	Nle	No	94	93	3,151.6	3,152.4
**7**	Dpr	Asn	Trp	Nle	No	99	99	2,407.3	2,408.5
**8**	Dpr(DOTA)	Asn	Trp	Nle	No	98	97	2,793.5	2,794.9
**9**	Lys	Lys	Trp	Nle	No	88	84	2,463.4	2,464.5
**10**	Lys(DOTA)	Lys(DOTA)	Trp	Nle	No	92	96	3,236.3	3,236.8
**11**	Lys (DOTA)	Asn	Trp	Nle	No	83^d^	83	2,834.5	2,835.3

^a^Percentage purity determined by HPLC using buffer system: *A *= TEAP (pH 2.5) and *B *= 60% CH_3_CN/40% *A *with a gradient slope of 1% *B*/min, at flow rate of 0.2 mL/min on a Vydac C_18 _column (0.21 cm × 5 cm, 5-μm particle size, 300-Å pore size). Detection at 214 nm. ^b^Percentage purity determined by capillary zone electrophoresis (CZE) using a Beckman P/ACE System 2050; field strength of 15 kV at 30°C. Buffer, 100 mM sodium phosphate (85:15, H_2_O:CH_3_CN), pH 2.50, on a Agilent μSil bare fused-silica capillary (75 μm i.d. × 40 cm length). Detection at 214 nm. ^c^MALDI mass spectral analysis (m/z). The observed [M + H]^+ ^of the monoisotopic mass (obs) compared with the calculated m/z of the monoisotope (calc). ^d^The detectable contaminant, produced during the dimerization process of this heterodimer, was identified as analog **3**.

**Table 2:** Binding affinities at NPY Y_1_- and Y_2_-receptors and Y_1_-related functional characteristics

Analog number	Binding affinity^a^		Functional assay
	Y_1 _	Y_2 _	cAMP for Y_1_
DOTA-free analogs			
**1**	11 ± 7	> 1,000	Antagonist
**3**	9.0 ± 3	> 1,000	Antagonist
**5**	143 ± 20	> 1,000	ND
**7**	19 ± 5	> 1,000	Antagonist
**9**	127 ± 50	> 1,000	Antagonist
DOTA-coupled analogs			
**2**	143 ± 37	> 1,000	Antagonist
**4**	294 ± 33	> 1,000	Antagonist
**6**	> 1000	> 1,000	ND
**8**	29 ± 7	> 1,000	Antagonist
**10**	283 ± 52	> 1,000	Antagonist
**11**	13 ± 3	> 1,000	Antagonist

Functional characteristics for free and DOTA-coupled dimeric peptide NPY analogs. ^a^IC_50 _values in nanomolars; mean ± SEM; *n *≥ 3. ND, not determined.

### Binding affinity profile

**Figure 3:**
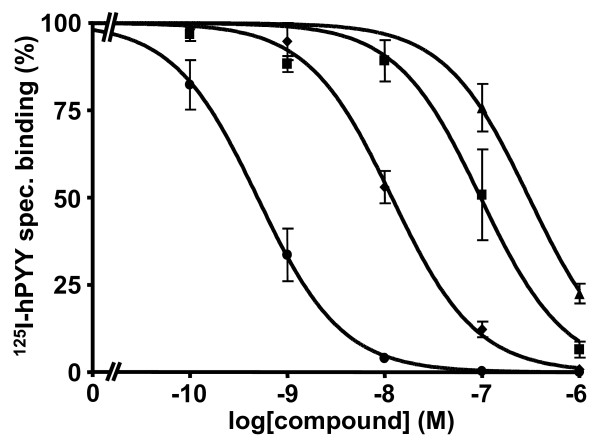
**Competition binding experiments using the NPY Y_1 _receptor-expressing SK-N-MC cell line**. All four tested compounds exhibit Y_1 _selectivity. While hPYY (solid circle) and **11 **(solid diamond) show high-affinity displacements of ^125^I-hPYY the analogs **9 **(solid square) and **10 **(solid triangle) show lower affinity displacements of ^125^I-hPYY. Dose response curves of at least three independent experiments ± SEM are shown.

The DOTA-free and DOTA-coupled analogs listed in Table 1 were analyzed in receptor autoradiography experiments for NPY Y_1 _and Y_2 _receptor-binding affinities on SK-N-MC cells endogenously expressing Y_1 _and SH-SY5Y cells endogenously expressing Y_2_, respectively (Table 2). Pharmacological displacement experiment using SK-N-MC cell membrane pellet sections for compounds **9**, **10**, and **11 **are shown in Figure 3. The IC_50 _values for all tested compounds are listed in Table 2. The addition of two DOTA moieties to the homodimeric analogs does decrease the Y_1 _binding affinity up to 2- to 30-fold. However, the addition of only one DOTA to heterodimer **7 **did markedly improve the binding affinity to an IC_50 _of 29 nM (**8**), as compared to the symmetric, bi-DOTA-linked parent scaffold (**6**) showing an IC_50 _over 1,000 nM. Finally, increasing the distance between the chelator and the peptide backbone, i.e., analog **11 **(Figure 2), enhanced the binding affinity (IC_50 _= 13 ± 3 nM; Table 2) of this heterodimer for the Y_1 _receptor. None of the tested compounds showed Y_2 _binding affinity.

### *In vitro *forskolin-stimulated adenylate cyclase activity

**Figure 4:**
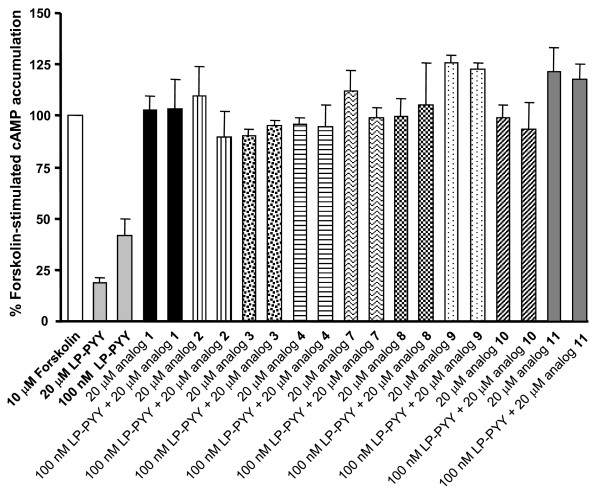
**Effect of various Y_1 _affine analogs on forskolin-stimulated intracellular cAMP accumulation in SK-N-MC cells**. Cells were incubated for 30 min with 10 μM forskolin either alone, or with 10 μM forskolin in the presence of 20 μM or 100 nM of the reference compound [Leu^31^, Pro^34^]-PYY (LP-PYY), or with 10 μM forskolin in the presence of the various Y_1 _affine analogs either alone at a concentration of 20 μM or at a concentration of 20 μM in the presence of 100 nM LP-PYY. Intracellular cAMP accumulation was then determined as described in Methods. Results are shown as percentage of the 10 μM forskolin effect on intracellular cAMP accumulation. While the 10 μM forskolin effect is efficiently inhibited by the agonist LP-PYY at 20 μM and 100 nM, all tested analogs behave as antagonist since given alone they are not able to inhibit the forskolin effect while they completely and efficiently antagonize the 100 nM effect of LP-PYY.

**Figure 5:**
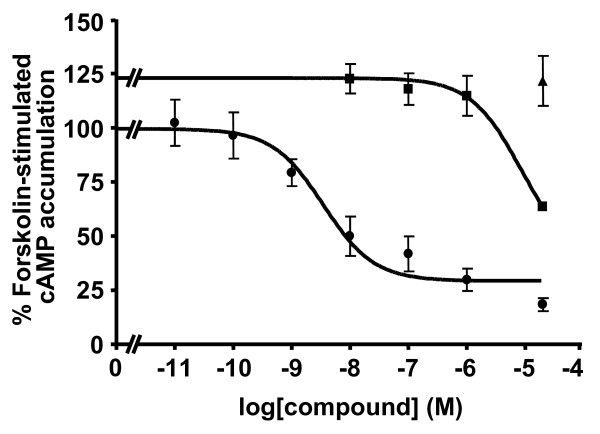
**Antagonistic effect of 11 on the inhibition of forskolin-stimulated intracellular cAMP accumulation in SK-N-MC cells**. Cells were incubated for 30 min with 10 μM forskolin in the presence of [Leu^31^, Pro^34^]-PYY (LP-PYY) at concentrations ranging between 0.01 nM and 20 μM alone (solid circle) or with 10 μM forskolin in the presence of LP-PYY at concentrations ranging between 0.01 nM and 20 μM supplemented with a fixed concentration of 20 μM of the analog **11 **(solid square). Compound **11 **behaves like an antagonist since it shifts the dose response curve of LP-PYY to the right. Compound **11 **given alone at a concentration of 20 μM has no effect on the accumulation of forskolin-stimulated cAMP (solid triangle).

Since the addition of a DOTA moiety can change the functional characteristics of a compound as recently shown in the somatostatin receptor field for sst_3 _[19], where a sst_3 _antagonist switched to an agonist upon the addition of a DOTA, compounds having a high or moderate Y_1 _affinity were analyzed in an adenylate cyclase activity assay for their agonistic or antagonistic properties. The results are shown in Figure 4. While the Y_1_-selective agonist [Leu^31^, Pro^34^]-hPYY, used as positive control, efficiently inhibited forskolin-stimulated cAMP accumulation when applied at concentrations of 20 μM and 100 nM, all tested compounds, DOTA-free and DOTA-coupled analogs behaved like full antagonists (Figure 4; Table 2). Given alone at a high concentration of 20 μM the analogs were not able to inhibit forskolin-stimulated cAMP accumulation but they efficiently antagonized the agonistic effect of 100 nM [Leu^31^, Pro^34^]-hPYY. Moreover, Figure 5 shows that 20 μM of **11**, the best compound of this series, given together with an increasing concentration of [Leu^31^, Pro^34^]-hPYY in the range from 10 nM up to 20 μM is able to shift by at least three orders of magnitude the dose response curve of [Leu^31^, Pro^34^]-hPYY to the right, indicating that **11 **efficiently antagonizes the agonist effect of [Leu^31^, Pro^34^]-hPYY. Thus, these DOTA-conjugated heterodimers, i.e., analogs **8 **and **11**, with their high binding affinity and antagonist property represent potential candidates for *in vivo *tumor targeting.

## Discussion

The recent demonstration that radiolabeled antagonists considerably improved the sensitivity of *in vivo *diagnostic procedures and might improve the efficacy of receptor-mediated radiotherapy suggested the generalized use of peptide antagonists rather than agonists for *in vivo *tumor detection [5,30]. However, selective peptide antagonists suitable for radiolabeling are not available for each receptor candidate and therefore they need to be developed. The high density and incidence of Y_1 _receptors in invasive and metastatic breast cancers, also expressing Y_2 _but not Y_4 _and Y_5 _receptors, make these neoplasms important targets for diagnosis and therapy with NPY-related drugs [8]. Thus, the aim of the present study was to design Y_1 _receptor affine antagonists suitable for molecular imaging.

As demonstrated, NPY exists in equilibrium between monomer and dimers in aqueous solution [31]. These dimeric structures were found to be not mandatory for the binding of NPY to Y_2 _receptors but were responsible for the increased Y_1_-affinity and selectivity. Proof of this concept was supported by the synthesis and the characterization of several dimeric NPY analogs retaining high Y_1_-affinity and selectivity over Y_2 _[23,25]. We thus derived a conceptual and experimental approach to the design of new dimeric Y_1 _receptor antagonists using the previously described dimeric scaffold, i.e., Bis(31/31')[Pro^30^, Cys^31^, Tyr^32^, Leu^34^]NPY(28-36)-NH_2 _(analog **1**), formed by the covalent linkage of two identical entities mimicking the C-terminal region of the full length NPY through a disulfide bridge between Cys moiety at position 31 [32]. As supported by a previous report, replacement of Tyr^32 ^by Trp and Leu^34 ^by Nle (**3**) did not produce a significant change in binding affinity or selectivity. However, adjunction of DOTA moieties to the N-termini of these dimeric peptides resulted in a dramatic loss of binding affinity (**2 **and **4**) suggesting the importance of the N-terminus for receptor recognition. In accordance, evidence demonstrating that N-terminal acylation might compromise receptor affinity and selectivity were already reported in the literature [33,34]. Such observation prompted us to evaluate different sites for the introduction of the DOTA. Thus, introduction of the DOTA moiety was achieved through its selective addition to the β or ε side chain amino group of a Dpr or Lys residue, respectively, introduced at position 29 which was found to be tolerant to amino acid replacement [25,26]. However, such substitution in each segment of the peptide dimers (**6 **and **10**) resulted in a loss of Y_1 _receptor-binding affinity compared to their DOTA-free counterparts (**5 **and **9**). Most likely, this reduction of affinity is probably due to the size and the negatively charged character of the DOTA moiety. As observed by NMR in BVD15, a monomer segment analogous to our peptide dimer, the Arg^33^, which was found to be particularly involved in the Y_1 _receptor recognition [27], and Asn^29 ^side chains seem to be spatially closed [35]. It is thus probable that coupling the DOTA moiety to the Dpr residue might disrupt the orientation or the character of the positively charged Arg residue and thus alter the overall binding affinity. Supporting this hypothesis, increasing the distance between the DOTA moiety and the peptide backbone, i.e., analogs **6 **and **10**, resulted in a less dramatic loss of binding affinity probably by reducing the electrostatic effect of the carboxyl groups of the DOTA moiety on the guanidinium derivative. The higher binding affinity of homodimeric NPY peptides was often related to the propensity of NPY receptors to form homodimers as recently demonstrated [36]. As such, by keeping intact one segment, original interaction between heterodimers (**8 **and **11**) and their receptor was restored; these two compounds being almost equipotent compared to the lead precursor (**1**). Although preliminary in nature, our results, and more particularly those of analog **11**, represent the first step towards the development of dimeric DOTA-coupled Y_1 _receptor antagonist for nuclear medicine application. Even if the stability of these analogs has not been evaluated in the present study, these compounds should prove to be sufficiently stable for tumor targeting purpose. Indeed, Bis(31/31')[Cys^31^, Nva^34^]NPY(27-36)-NH_2_, presenting a high sequence and scaffold homology with our compounds was able to stimulate *in vivo *the food intake in rats in a similar manner than that observed by other non-dimeric Y_5 _selective agonists [37].

In analogy to somatostatin receptor targeting of tumors, it has been proposed to use NPY analogs to target NPY receptors for tumor therapy. NPY analogs suitable for this purpose have indeed already been developed, such as a daunorubicin-coupled cytotoxic NPY analogs [16] and a Y_2_-selective ^99 m^Tc-labeled radioactive NPY analog [17], all being agonists. Assuming that the observation of the superiority of somatostatin receptor antagonists, but also of bombesin receptor antagonists [30] for tumor targeting can be generalized, the present DOTA-coupled high-affinity Y_1 _receptor antagonist may be a useful tool for the diagnostic and radiotherapeutic targeting of Y_1_-expressing tumors. Breast tumors with their high Y_1_-receptor density would represent first choice candidate tumors. Other tumor types, such as renal cell carcinomas, ovarian cancers, adrenal tumors and embryonic tumors, may also be targets of interest. The same general principles as for somatostatin receptor targeting could be applied. Advantages that should be put forward are a more favorable benefit-toxicity profile compared with conventional radio- or chemotherapy and the rarity of side effects. The radiotargeting of NPY receptor-expressing tumor blood vessels alone or together with NPY receptor-expressing tumor cells may also represent an attractive strategy for therapy. Finally, since many of the NPY receptor-expressing tumors can express multiple peptide receptors concomitantly, NPY receptors may be suitable for a multireceptor targeting with a cocktail containing NPY and other therapeutic peptide analogs directed against various peptide hormone receptors. For such a multireceptor approach, good candidate tumors seem to be breast tumors targeted with NPY and bombesin analogs.

## Conclusions

In the course of designing and synthesizing DOTA-coupled dimeric NPY Y_1 _receptor antagonists, we found that the addition of a DOTA moiety to both peptide segments negatively influenced the binding affinity of all dimeric compounds synthesized and that the asymmetric introduction of a DOTA to one of the segments of the peptide heterodimer yields compounds exhibiting high binding affinity and Y_1_-selectivity over Y_2_. Analogs **8 **and **11 **are the first dimeric high-affinity DOTA-coupled Y_1 _receptor antagonists. They may, when linked to an adequate radiometal, become useful tools for *in vivo* tumor targeting of Y_1_-positive tumors, particularly breast tumors.

## Abbreviations

The abbreviations for the common amino acids are in accordance with the recommendations of [38]. Additional abbreviations: Acm: acetamidomethyl; Boc: *tert*-butoxycarbonyl; BSA: bovine serum albumin; cAMP: 3',5'-cyclic adenosine monophosphate; ^14^C: ^14^Carbon; CZE: capillary zone electrophoresis; DCM: dichloromethane; DIC: N, N'-diisopropylcarbodiimide; DIPEA: N, N'-diisopropylethylamine; DMF: dimethylformamide; DMSO: dimethylsulfoxide; DOTA: 1,4,7,10-tetraazacyclododecane-1,4,7,10-tetraacetic acid; Dpr: 2,3-diaminopropionic acid; FBS: fetal bovine serum; Fmoc: 9-fluorenylmethoxycarbonyl; IBMX: 3-isobutyl-1-methylxanthine; ^125^I: ^125^Iodine; MALDI-TOF-MS: matrix assisted laser desorption/ionization-time off light mass spectrometry; MBHA: 4-methylbenzhydrylamine; MEM: minimum essential medium; NHS: N-hydroxysuccinimide; NMR: nuclear magnetic resonance; Nle: norleucine; NPY: neuropeptide Y; Nva: norvaline; PP: pancreatic polypeptide; PYY: peptide YY; RP-HPLC: reversed-phase high-performance liquid chromatography; TEA: triethylamine; TEAP: triethylammonium phosphate; TFA: trifluoroacetic acid.

## Competing interests

The authors declare that they have no competing interests.

## Authors' contributions

DC: carried out the synthesis of the peptides and participated in the manuscript draft.

RC: carried out the functional assays.

BW: carried out the binding assays.

JE: carried out the synthesis of the peptides and reviewed the chemistry data. JER: participated in the conception, design and coordination of the study, and reviewed the chemistry data.

JCR: participated in the conception, design and coordination of the study, and in the interpretation of the data. He reviewed the draft manuscript.

All authors read and approved the final manuscript.
